# Neuronal Nicotinic Receptors as New Targets for Amphetamine-Induced Oxidative Damage and Neurotoxicity

**DOI:** 10.3390/ph4060822

**Published:** 2011-06-15

**Authors:** David Pubill, Sara Garcia-Ratés, Jordi Camarasa, Elena Escubedo

**Affiliations:** Unitat de Farmacologia i Farmacognòsia, Facultat de Farmàcia, Universitat de Barcelona, 08028 Barcelona, Spain; E-Mails: sgarcira@gmail.com (S.G.-R.); jcamarasa@ub.edu (J.C.); eescubedo@ub.edu (E.E.)

**Keywords:** MDMA, methamphetamine, nicotinic, neuroprotection, calcium influx

## Abstract

Amphetamine derivatives such as methamphetamine (METH) and 3,4-methylenedioxymethamphetamine (MDMA, “ecstasy”) are widely abused drugs in a recreational context. This has led to concern because of the evidence that they are neurotoxic in animal models and cognitive impairments have been described in heavy abusers. The main targets of these drugs are plasmalemmal and vesicular monoamine transporters, leading to reverse transport and increased monoamine efflux to the synapse. As far as neurotoxicity is concerned, increased reactive oxygen species (ROS) production seems to be one of the main causes. Recent research has demonstrated that blockade of α7 nicotinic acetylcholine receptors (nAChR) inhibits METH- and MDMA-induced ROS production in striatal synaptosomes which is dependent on calcium and on NO-synthase activation. Moreover, α7 nAChR antagonists (methyllycaconitine and memantine) attenuated *in vivo* the neurotoxicity induced by METH and MDMA, and memantine prevented the cognitive impairment induced by these drugs. Radioligand binding experiments demonstrated that both drugs have affinity to α7 and heteromeric nAChR, with MDMA showing lower K_i_ values, while fluorescence calcium experiments indicated that MDMA behaves as a partial agonist on α7 and as an antagonist on heteromeric nAChR. Sustained Ca increase led to calpain and caspase-3 activation. In addition, modulatory effects of MDMA on α7 and heteromeric nAChR populations have been found.

## 1. Introduction

**Figure 1:**
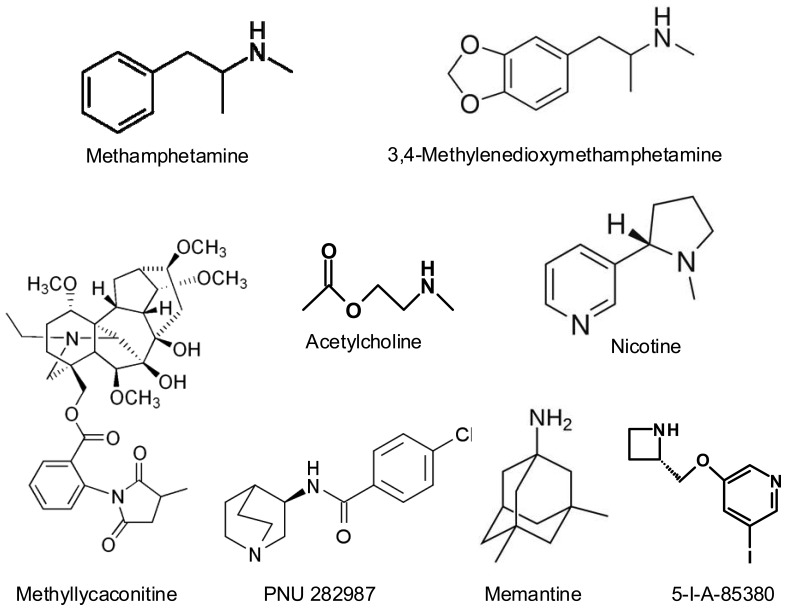
Chemical structures of the main compounds cited in this article.

Methamphetamine (METH) and 3,4-methylenedioxymethamphetamine (MDMA, “ecstasy”) are amphetamine derivatives (Figure 1) that are widely abused in developed countries, due their potent stimulating effects on the central nervous system. They are usually taken in a recreational context, producing feelings of euphoria, well-being and connectedness with others, enhancing sensitive perceptions and personal relationships. The widespread use of these drugs has led to concern because of the extensive evidence that they are neurotoxic in animal models (for reviews, see [1-4]). The neurotoxic consequences of the acute or long-term use of these substances in humans are still uncertain and a great deal of research is being done on the subject. In fact, cognitive impairment and dopaminergic/serotonergic deficits have been described in chronic abusers of these drugs [5-10]. A recent meta-analysis on the effects of MDMA on short-term and working memory [11] concludes that ecstasy users perform significatively worse in all memory domains, both in studies using drug-naïve controls and studies using polydrug controls, and that this impairment is related to total lifetime ecstasy consumption. It is a consistent finding that ecstasy users display significantly more psychopathology than non-users. The relationship is the most evident for depression, anxiety, phobias, psychotic symptoms, somatization, aggression, hostility, impulsiveness and sensation seeking behaviour (reviewed by [5]). However, the results of these studies are biassed by the fact that it is practically impossible to find exclusive MDMA consumers, but it is taken associated with other drugs such as cannabis, cocaine, alcohol, *etc.*, which can modify its psychiatric effects.

Long-term damage to dopaminergic and serotonergic nerve terminals after chronic abuse of METH and MDMA has been reported by a number of preclinical studies in several brain areas. Concretely, treatment with METH induces long-lasting depletion in the striatal content of dopamine (DA) and its metabolites [12], decrease in tyrosine hydroxylase activity [13,14] and loss of dopamine transporters (DAT) [15,16]. On the other hand, the deleterious effects of MDMA have been found to affect more specifically serotonergic terminals in rats and primates, with little effect on dopaminergic terminals [17,18]. Conversely, MDMA behaves as a dopaminergic neurotoxin in mice, with additional serotonergic depletions [19].

From the first time it was reported, the study of the mechanisms of neurotoxicity induced by amphetamine derivatives has generated an important amount of works. Although some aspects are still awaiting an explanation, there is a clear evidence of the coordinate action of several key phenomena that contribute to such effects, namely vesicular (VMAT-2) and plasmalemmal dopamine transporters (DAT) function, mitochondria and energy balance, glutamate, dopamine receptors, hyperthermia and reactive oxygen species (ROS) (reviewed by [20]). Since the role of ROS was proposed by Gibb and co-workers in 1989 [21], subsequent works have demonstrated that these species seem to be the final executors of neuronal damage, reacting with functional and structural molecules and inducing degenerative changes. ROS can originate from the auto-oxidation of cytosolic DA [22], glutamate excitotoxiciy leading to mitochondrial dysfunction and nitric oxide (NO) production [23,24], activation of D1 receptors within the striatum leading to increases in nNOS mRNA expression [25]; and inhibition of mitochondrial function increasing mitochondrial-mediated ROS generation. Also, activation of microglia (source of reactive species) has been reported after METH treatment [15,17,26,27] but not after MDMA [17]. Additionally, a role of a metabolic reactive derivative of MDMA in the neurotoxic process has been proposed [28].

According to the ROS hypothesis an *in vitro* model using rat striatal synaptosomes was set up to induce and detect the production of these species using flow cytometry and a ROS-sensitive fluorescent probe [29]. This provided a system where the participation of several signalling pathways in ROS production could be studied. The fact that the nicotinic acetylcholine receptor (nAChR) antagonist methyllycaconitine (MLA) blocked METH-induced ROS in this model pointed to a new mode of action of amphetamines that deserved further research. In this article we will review and integrate all the evidence concerning the role of neuronal nicotinic receptors in the mode of action of amphetamine derivatives.

## 2. Some Generalities about nAChR

Neuronal nAChR belong to the superfamily of ionotropic receptors and include a number of subtypes formed by the association of five subunits encoded by different genes. To date, the genes that have been cloned include two subfamilies of nine α (α2-α10) and three β (β2-β4) subunits and are expressed in the nervous system, cochlea and a number of non-neuronal tissues [30-32]. nAChR subunits assemble in pentamers which can be homomeric or heteromeric, forming a central ion pore with different structural, functional and pharmacological properties [33]. Two main classes have been identified: the α-bungarotoxin (α-BgTx)-sensitive receptors, which are made up of the α7, α8, α9 and/or α10 subunits and can form homomeric or heteromeric receptors, and α-BgTx-insensitive receptors that consist of α2-6 and (β2-4) subunits, and bind nicotine and many other nicotinic agonists with high affinity but not α-BgTx [34].

Depending on their subunit composition nAChRs are permeant to the cations Na^+^ and K^+^or Ca^2+^ (reviewed in [35]). Thus heteromeric nAChR made of α and β subunits have in general a low permeability for Ca^2+^ (fractional current of 2-5%). By contrast, homomeric α7 subtypes have the highest fractional Ca^2+^ current, which ranges from 6% to 12% depending on the species. An important issue is the fact that the fractional Ca^2+^ current through human α7 nAChR is the highest reported for homomeric ligand-gated receptors, matching that of heteromeric NMDA receptors [36]. Also, depolarisation induced by entry of Na^+^ or Ca^2+^ could induce voltage-gated-calcium channels opening and enhance Ca^2+^ influx. These two mechanisms can be physiologically complementary and play important roles in cell signalling by activating different downstream intracellular neuronal pathways (reviewed in [37]) such as protein kinase C (PKC) and neuronal nitric oxide synthase (nNOS), which have similarly been implicated in the neurotoxicity of amphetamines [38,39].

nAChR have a number of allosteric binding sites in addition to the ACh binding sites. Thus several compounds with different chemical structures have been found to bind to these sites and behave as allosteric modulators of nAChR function (reviewed in [40]).

The brain functions were nAChR play a role include cognition, locomotion and analgesia [41-44] and nicotine addiction [45]. In the CNS nAChR are mainly located presynaptically modulating the release of almost all neurotransmitters, including dopamine, but also have a post-synaptic localization in some areas, where they mediate fast synaptic transmission [34,37,40].

## 3. Role of nAChR in METH- and MDMA-Induced ROS Production

### 3.1. ROS and Amphetamine Neurotoxicity

The preponderant role of ROS in METH- and MDMA-induced neurotoxicity has been extensively demonstrated by the fact that inhibition of their formation or preventing their action affords neuroprotection against these substances. Thus, enhancement of the antioxidant resources of the cells such as glutathione peroxidase [46] or isoforms of superoxide dismutase [47,48] is neuroprotective against amphetamine derivatives. Also, antioxidants as selenium and melatonin, [49,50], L-carnitine [51], N-acetylcysteine [52] or the endogenous antioxidant carnosine [53] are also neuroprotective against this damage.

In addition, amphetamine derivatives induce *in vivo* a significant increase in body temperature which is potentiated by a high ambient temperature [53], as a consequence of their central thermo-disregulatory effect. It has been suggested that hyperthermia might also potentiate the production of 6-hydroxydopamine or related ROS after drug exposure [54] or potentiate the propagation of ROS damage to lipids. Thus hyperthermia potentiates amphetamines' neurotoxicity [55], and it has been demonstrated that reducing hyperthermia attenuates long-term decreases in DA and 5-HT content [56]. For this reason it was lately found that the neuroprotective effects afforded by some drugs was due to their reduction of hyperthermia rather than to an effect on its main pharmacological target [57,58]. Therefore it was necessary to set up an *in vitro* model where ROS production could be induced and the interferences of an antihyperthermic effect could be avoided. This allowed testing the role of determined pathways in METH- and MDMA-induced ROS production without such interferences.

### 3.2. Mechanisms Involved in ROS Production Induced by METH and MDMA in Striatal Synaptosomes

The effect of METH and MDMA addition on ROS production was studied using a preparation of semi-purified rat striatal synaptosomes loaded with the ROS-sensitive fluorescent probe 2′,7′-dichlorofluorescin diacetate (DCFH-DA) and a flow cytometer equipped with an argon laser as the measurement instrument [29,79].

**Figure 2:**
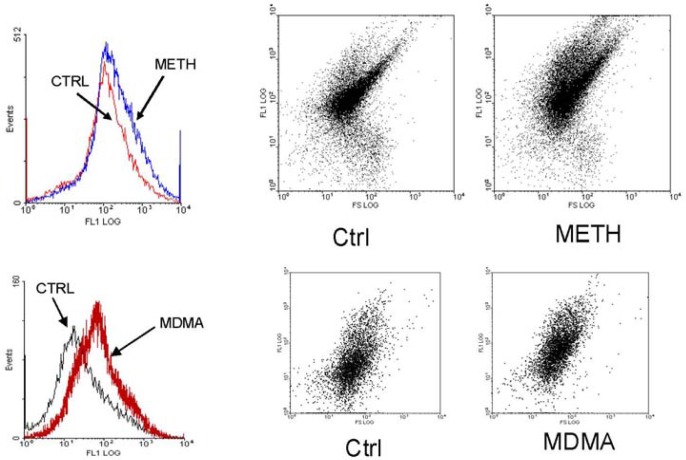
Representative fluorescence flow cytometry histograms and dot plots depicting the increase in ROS (DCF fluorescence) induced by previous incubation of rat striatal synaptosomes with METH or MDMA [29,79]. The histograms shift to the right (higher fluorescences) as well as individual synaptosomes show higher fluorescence intensity.

METH increased DCF fluorescence when added to synaptosomes, indicating that it induces ROS production in neuron terminals (Figure 2). This increase was measured inside the synaptosomes and not in the medium, due that flow cytometer measures the fluorescence associated with each particle. It has been established that acute incubation of synaptosomes with METH causes release of dopamine from presynaptic nerve terminals and inhibits DA uptake, probably by reversion of DAT functionality [59]. Released DA can undergo oxidation and generate ROS but, as only the fluorescence associated with synaptosomes was measured; this putative source of ROS was not taken into account. Moreover, if ROS formation took place mainly in the extracellular medium this would not explain the specificity of METH degeneration for DA terminals, in that the oxidation of extraneuronal DA would be expected to nonspecifically damage all neighboring neurons, not just the dopaminergic. Thus, this model allowed describing an intracellular oxidative effect of METH that was more likely to induce selective damage to dopamine terminals.

Several authors point to dopamine as one of the main sources of ROS induced by amphetamines [60,61]. When synaptosomes from previously DA-depleted rats (treated with reserpine or reserpine plus α-methyl-*p*-tyrosine) were tested the METH-induced ROS production was inhibited [29]. These results corroborate DA as the main source of the measured ROS. METH, by altering the intracellular pH gradient, prevents the function of the vesicular monoamine transporter (VMAT) and promotes DA release from vesicles to cytosol [62] where it can be oxidized. Accordingly, *in vitro* incubation of non-depleted synaptosomes with substances that block VMAT (reserpine) prevented METH oxidative effect [29].

Nitric oxide (NO) and peroxynitrite (ONOO^−^) are two reactive species that play a key role in the neuronal damage induced by amphetamine derivatives. A number of *in vivo* studies demonstrate the involvement of neuronal nitric oxide synthase (nNOS) in METH neurotoxicity. Thus, METH administration causes overexpression of nNOS in mouse striatum [38]. NO is an inhibitor of mitochondrial respiratory chain complexes II and IV and rapidly reacts with superoxide to yield ONOO^−^, which is a powerful oxidant and a potentially irreversible inhibitor of complexes II and III. The ONOO^−^ formed inside mitochondria impairs mitochondrial functions and integrity. Also ONOO^−^ oxidises glutathione, α-tocopherol and ascorbate, thereby compromising essential antioxidant pools within mitochondria [63,64]. NO and Ca^2+^ synergistically inactivate mitochondrial complex I and cause a loss of cytochrome c, probably via formation of ONOO^−^ [65].

In this striatal synaptosomes model, the inhibitor of nNOS, 7-nitroindazole, completely abolished METH-induced ROS production, demonstrating a role of this enzyme in METH oxidative effects. Activation of nNOS produces NO, which reacts with the peroxide radicals which would originate from DA autooxidation, producing the more toxic radical peroxynitrite. This oxidant has been found to inhibit DAT functionality [66,67]. Such an inhibition would favour cytosolic DA accumulation, which would increase oxidative species inside the synaptosomes. Peroxynitrite has been postulated as the main species responsible for the damage in cell structures [66]. Accordingly, inhibition of NO formation through a variety of methodological approaches has confered neuroprotection against METH or MDMA: nNOS deficient mice are resistant to METH-induced dopaminergic neurotoxicity [68]; administration of NO synthase inhibitors such as S-methylcitrulline or AR-R17477 attenuates the dopaminergic and serotonergic neurotoxicity of MDMA and METH [69-73]. Finally, selenium (more effective as a scavenger of two-electron oxidants, such as ONOO^−^ and not particularly reactive towards single electron oxidants, such as NO and superoxide) shows a high neuroprotective effect against METH-induced neurotoxicity [74].

Kinases such as protein kinase C (PKC) have been implicated in various aspects of DAT function and direct phosphorylation [75]. It has been described that PKC contributes to DAT phosphorylation producing an impairment of its function [76-78]. Accordingly, in the synaptosomes model, inhibition of PKC fully prevented METH-induced ROS [29], corroborating a key role of PKC in this process. The prevention by the PKC inhibitor could be explained by the maintenance of DAT function, which would be capable of releasing the increased cytoplasmatic dopamine to the extracellular medium, thus avoiding its conversion to ROS inside the terminal.

Similar results as above have been obtained with METH and MDMA on mouse striatal synaptosomes [79,80]. The concentration-response curve of MDMA showed an inverted U-shape, with maximal pro-oxidative activity between 50 and 100 μM, declining at higher concentrations. An explanation for this effect is given by the fact that, at concentrations above 100 μM, MDMA inhibits monoamine oxidase B (MAO-B), which is responsible for enzymatic dopamine degradation generating hydrogen peroxide. In fact, pharmacological inhibition of MAO-B by L-deprenyl abolished the oxidative effect of 50 μM MDMA.

Taken together, in the synaptosomes model an increase in cytosolic DA and activation of nNOS and PKC (blocking DA transport through DAT) are needed to generate ROS inside the dopaminergic terminal. Moreover, both PKC and nNOS are enzymes that require calcium to be activated. Consequently, when calcium of the medium is chelated with EGTA, the oxidative effect of METH and MDMA is prevented in this model.

To sum up, an integrative mechanism by which METH/MDMA induce ROS production in striatal synaptosomes was postulated: the drug enters the synaptosome, either by passive diffusion or through DAT, as in the case of MDMA (cocaine, a DAT blocker, prevented MDMA but not METH oxidative effect [81]), and promotes DA release from synaptic vesicles to the cytosol. Increased cytosolic DA can suffer from autoxidation and generate initial ROS which can modify DAT function. Additionally, there is an increase in intrasynaptosomal Ca^2+^, which would activate nNOS and PKC. PKC activation would lead to phosphorylation of proteins such as DAT promoting, together with ONOO^−^, a reduction of DAT activity and accumulation of cytosolic DA that would impair the initial oxidative stress.

Looking at this hypothesis, a key point remained to be addressed: the mechanism by which METH and MDMA increased cytosolic calcium. The L-type voltage-gated calcium channel blocker, nitrendipine, and the inhibitors of calcium release from the endoplasmic reticulum, dantrolene and 2-aminoethoxydiphenylborate (2-APB), inhibited MDMA-induced ROS in mouse striatal synaptosomes [81]. This, in agreement with extracellular Ca^2+^ chelation experiments, suggested that there had to be a preceeding depolarizing event induced by METH/MDMA that triggered the opening of voltage-gated channels or calcium-induced calcium release from the endoplasmic reticulum.

Liu *et al.* [82] had reported that D-amphetamine enhanced Ca^2+^ entry and catecholamine release in bovine adrenal chromaffin cells *via* the activation of a nicotinic receptor resembling the α7 subtype. In addition, Skau and Gerald [83] had described that D-amphetamine inhibits α-bungarotoxin binding at the neuromuscular junction in mice, while Klingler *et al.* [84] more recently identified nAChR as one of the physiological targets of MDMA in the neuromuscular junction. This led to test the α7 nAChR antagonist methyllycaconitine (MLA) and α-bungarotoxin on METH- and MDMA-induced ROS production in striatal synaptosomes. Both antagonists conferred protection against ROS production in our model [29,80], pointing to α7 nAChR as a putative new target to prevent amphetamines' oxidative damage.

## 4. nAChR Modulate the Effects of METH and MDMA on Dopamine Transporters

Acute treatment with METH or MDMA in rats induces an impairment of the uptake of radiolabeled neurotransmitters (*i.e.*, dopamine and serotonin, 5-HT) in striatal synaptosomes, as a consequence of the rapid and reversible changes induced by these drugs on monoamine transporters. This effect can be reproduced by incubating *in vitro* the synaptosomes [77] with the drugs. For this reason the synaptosomal model described can be used to study the possible modulation of these effects by nicotinic ligands.

[^3^H]5-HT uptake (in hippocampal) and [^3^H]DA uptake (in striatal synaptosomes) were measured as indicative of the acute serotonergic effect of MDMA and the acute dopaminergic effect of METH, respectively [29,79,80,88]. Preincubation of synaptosomes with MDMA (15 μM) induced a significant reduction in [^3^H]5-HT uptake by 40%. MDMA (10 μM) and METH (1 μM) also inhibited [^3^H]dopamine uptake by 75% and 80% respectively. This inhibition remained even after drug washout and therefore cannot be attributed to residual drug presence but to a persistent alteration of transporters. As incubation of drugs with synaptosomes was carried out in the presence of glutathione and a MAO-A inhibitor (chlorgiline), the effect of these drugs on monoamine transporters cannot be attributed to ROS.

The effect of the amphetamine derivatives on these transporters was prevented by calcium chelation and inhibition of nNOS and PKC (both calcium-dependent enzymes). Some authors had already described the relationship between PKC and the effect of amphetamines on DAT [77]. Additionally, the physical association of nNOS and serotonin transporter (SERT) has been reported, resulting in modulation of SERT activity [85]. Also, Cao and Reith [86] described how NO inhibits DA uptake.

Based on the results reported on ROS production, the α7 nAChR antagonists MLA and memantine were tested in this model [29,79,80], and found to prevent the effects of METH and MDMA on DAT. Memantine (see section 6 for further details on this drug) has a dual mechanism as glutamate NMDA receptor antagonist and as an α7 nAChR antagonist [91]. PNU 282987 (an α7 nAChR agonist) prevented the protective effect of MEM, but MK-801 (glutamate NMDA receptor antagonist) did not modify it, confirming that the effect of MEM on MDMA/METH-induced uptake inhibition is mediated by α7 nAChR and not by blockade of NMDA receptor. These results suggest that activation of nAChR alone or combined with other effects of amphetamine derivatives leads to the activation of pathways (*i.e.*, nNOS or PKC) involved in monoamine transporter inhibition.

Aznar *et al.* [87] described the presence of α7 nAChR at serotonin neurones, in terminals projecting to the hippocampus. Accordingly, PNU 282987 alone inhibited 5-HT uptake, which suggests that SERT functionality in the hippocampal serotonergic terminals can be regulated by α7 nAChRand potentiated the inhibitory effect of MDMA on SERT function [88,89].

## 5. The α7 nAChR Antagonist MLA Protects *In Vivo* against METH and MDMA Neurotoxicity

The next step was to assess whether the protective effects observed *in vitro* had an *in vivo* translation preventing or attenuating the amphetamine-induced damage. For this reason animal experiments were conducted treating mice with a classic neutoxic dosing schedule of METH (7.5 mg/kg s.c., every 2 h, for a total of four doses) or MDMA (25 mg/kg, s.c., every 3 h, for a total of three doses) and compared some of the main neurotoxicity markers with those from mice that had previously received MLA [79,80]. In both cases, an antihyperthermic effect of MLA was ruled out.

METH induced, at 72 h post-treatment, a significant loss of striatal DA reuptake sites of about 73%, measured as specific binding of [^3^H]WIN 35428 in mouse striatum membranes [79,80]. This dopaminergic injury was attenuated in mice pretreated with MLA (from 73% to 43%) without affecting METH-induced hyperthermia. Also, MLA prevented the decrease in tyrosine hydroxylase, the key enzyme in dopamine synthesis whose loss is also correlated with dopaminergic impairment. Moreover, pretreatment with MLA prevented the striatal inflamatory glial activation assessed 24 h after treatment as an increase in [^3^H]PK 11195 specific binding.

**Figure 5:**
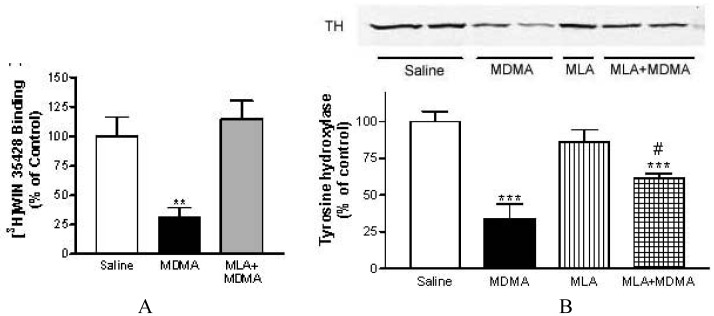
Protective effects of MLA on striatal dopaminergic neurotoxicity in mice treated with MDMA. The loss of dopamine transporters was quantified by [^3^H]WIN 35428 binding (panel A) and the dopamine impairment was measured by Western blotting (panel B) using a primary antibody against tyrosine hydroxylase (reproduced from [79]).

Similar results were obtained with MDMA and MLA (Figure 5). Surprisingly, MLA did not prevent the loss in [^3^H]paroxetine binding sites indicating that its neuroprotective effect in mice is selective for dopaminergic terminals [80]. This selective dopaminergic neuroprotection of MLA has been corroborated more recently by other researchers [90].

## 6. Memantine, a Drug Used in Alzheimer's Disease, Is also an α7 nAChR Antagonist and Protects *In Vivo* against METH and MDMA Neurotoxicity

Memantine (MEM) is a non-competitive antagonist of the NMDA glutamate receptor that is currently being used to treat moderate-to-severe Alzheimer's disease. It possesses voltage-dependent binding properties that confer the ability of reducing tonic (excytotoxic), but not synaptic, NMDA receptor activity. In addition it was found that MEM, at clinically relevant concentrations, blocks α7 nAChR in a non-competitive manner, even more effectively that it does at NMDA receptors [91]. Accordingly, Unger *et al.* [92] had described how treatment with MEM significantly increases the number of α7 nAChR binding sites in frontal and retrosplenial cortex in mice, suggesting the interaction of MEM with these nicotinic receptors, as up-regulation of nAChR is a characteristic effect induced by nicotinic ligands (agonists and antagonists, see point 9 for further information).

The use of MLA as a medicine in humans could be precluded by its chemical complexity and toxic side effects [93,94]. By contrast, if MEM, due to its dual mechanism, prevented METH and MDMA-induced neurotoxicity in rodents, it could be proposed as a treatment in humans to prevent the effects of these amphetamine derivatives or even to treat addiction. Moreover, it might also have a beneficial effect on the memory impairment that abusers of these drugs usually suffer from [95,96].

Accordingly the effect of MEM on dopaminergic neurotoxicity (characteristic of METH and MDMA in mice) was studied using the *in vitro* striatal synaptosomes model [88,89]. MEM had not direct antioxidant properties against H_2_O_2_ but, at low (0.3 μM) micromolar concentrations, inhibited the ROS production induced by MDMA and METH. This inhibition was countered by the presence of PNU 282987, a specific agonist of the α7 nAChR, and was not modified by glutamate agonists in the incubation medium, indicating that the protective effect took place through α7 blockade.

*In vivo* experiments were conducted administering MDMA to Dark Agouti rats (18 mg/kg, s.c.) as a model of serotonergic neurotoxicity induced by MDMA [88,89]. Dark Agouti rats (a strain devoid of some CYP isoforms) suffer a significant serotonergic lesion in response to just a single dose of MDMA [97], conversely to what occurs with the more usual strains, needing several doses to show a similar injury [98]. Studies in mice adminsitered with METH were also carried out to study the effect of MEM on dopaminergic METH-induced neurotoxicity. MEM (5 mg/kg, i.p.) was administered 30 min before the corresponding dose of MDMA or METH and did not modify the hyperthermic response in any of the cases.

**Figure 6:**
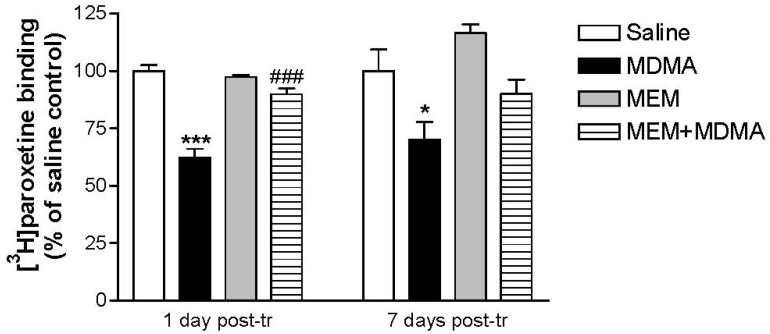
Prevention by memantine of the loss of serotonergic terminals induced by MDMA [89]. Dark Agouti rats were treated with a neurotoxic dose of MDMA plus/minus memantine (MEM) and killed 1 or 7 days after. [^3^H]paroxetine binding was performed in hippocampal membranes. ANOVA: * P < 0.05, *** P < 0.001 *vs.* saline; ### P < 0.001 *vs.* MDMA.

A significant decrease in the density of serotonin transporters (assessed by [^3^H]paroxetine binding) was observed in the hippocampus and frontal cortex of MDMA-treated rats killed 7 days post-treatment, although such a serotonergic injury was already apparent 24 h post-treatment [89]. In both cases, MEM significantly prevented the loss of binding sites, suggesting a neuroprotective effect on serotonergic terminals (Figure 6). MEM also prevented the delayed glial activation that was detected as an increase in [^3^H]PK 11195 binding in the animals killed 7 days after treatment, supporting the protective effect.

Different transcription factors such as the nuclear factor kappa B (NF-kB) can be activated after increased ROS production. NF-kB induces the expression of pro-inflammatory and cytotoxic genes and plays a key role in the balance between cell survival and death. The translocation (from cytosol to the nucleus) of P65, the active subunit of NF-kB, was measured in the hippocampus of differently treated rats, detecting a significant increased p65 nuclear translocation in the hippocampus of MDMA-treated animals, which indicates activation of NF-κB. P65 translocation was inhibited by MEM pretreatment; suggesting that activation of NF-kB after treatment with MDMA participates in the cytotoxic effect, since when this activation is blocked by MEM, neuronal injury is prevented. As for dopaminergic damage is concerned, MEM also prevented the loss in [^3^H]WIN 35428 binding and tyrosine hydroxylase, as well as the microglial response [89].

MEM showed a better protective effect in front of MDMA- and METH-induced neurotoxicity than MLA, which is a more specific α7 nAChR antagonist. Although MEM could directly prevent the MDMA- and the METH-induced neurotoxicity through antagonism at NMDA receptors, this is not a feasible hypothesis since antagonists of these receptors fail to prevent the oxidative stress and cell death induced by METH and MDMA [99]. However, the dual antagonism that MEM exerts on NMDA receptor and on α7 nAChR probably turns it into a better pharmacological tool to prevent amphetamines-induced damage *in vivo*. Firing of dopamine neurons is modulated by glutamatergic (excitatory) afferents and DA release is evoked by NMDA. If MEM blocks NMDA receptors, a decrease in extracellular DA levels would take place. Following the integrated hypothesis by Sprague *et al.* [100], the probability that released DA might be taken up into the depleted 5-HT terminals would be reduced by MEM. Consequently, the antagonism at NMDA receptors could contribute to the protective effects of MEM. Moreover, it has been recently reported that glutamate release induced by METH is abolished by MLA, indicating that it is triggered by α7 nAChR activation [90]. Therefore memantine would inhibit both glutamate release and NMDA receptor activation, showing enhanced neuroprotective properties when compared with MLA.

## 7. Memantine Prevents the Cognitive Impairment Induced by METH and MDMA

Having observed the neuroprotective effects of MEM, the next step was to investigate whether this drug could prevent the cognitive deficits induced by the amphetamine derivatives. There were carried out experiments to demonstrate a specific effect of MDMA or METH treatment and the possible modulation by MEM on the object recognition memory test and the Morris water maze, using Long Evans rats [101,102]. The animals pre-treated with MEM did not show the memory impairment that appeared in MDMA- or METH-treated animals. Therefore MEM, by preventing MDMA or METH-induced neuronal injury, contributes to attenuate the cognitive impairment produced by amphetamine derivatives. This preventive effect on memory impairment suggests a novel therapeutic approach to the treatment of CNS long-term adverse effects of amphetamine derivatives.

## 8. Amphetamine Derivatives Directly Interact with nAChR

Looking at the effects found *in vitro* and *in vivo* it was necessary to investigate if METH and MDMA had affinity for nAChR. For this reason radioligand binding experiments were carried out using [^3^H]epibatidine to label heteromeric receptors and [^3^H]MLA to label homomeric α7. METH and MDMA displaced both [^3^H]epibatidine and [^3^H]MLA binding in PC12 cells and mouse brain membranes, indicating that they can directly interact with nAChR [103]. MDMA displayed higher affinity than METH for both subtypes of nAChR. The resulting K_i_ values fell in the micromolar range, some in the low micromolar range and others in the high micromolar range (Table 1).

Special attention must be paid in the affinity for heteromeric receptors (K_i_ about 0.7 μM) which is practically the same that the K_i_ displayed by MDMA for the serotonin transporter, its main physiological target (0.61 μM) [98]. Therefore an interaction of MDMA on heteromeric nAChR at recreational doses is certainly possible. The fact that the lowest K_i_ values were found against [^3^H]epibatidine binding indicates that METH and MDMA displayed higher affinity for heteromeric nAChR which are the most abundant in the CNS. Also, similar results were found in rat brain membranes in which the K_i_ of MDMA and METH for α7 nAChR was around 9 and 4 μM, respectively.

## 9. METH and MDMA Induce Up-Regulation of Nicotinic Receptors

After prolonged contact with an agonist (*i.e.*, nicotine) nAChR exhibit a particular regulation: contrarily to what is generally expected after continuous stimulation, a down-regulation, these receptors develop an increase in ligand binding (up-regulation) [104,105]. A number of works have been focused in the study of the complex mechanisms involved in such up-regulation of nAChR (reviewed by [106]). nAChR up-regulation could be a response to the desensitization that follows the constant presence of an agonist [107], in order to restore necessary nicotinic transmission.

The mechanism through which nicotine induces nAChR up-regulation is complex and not fully clarified to date. There are reports indicating that nicotine-induced increases in nAChR are not accompanied by changes in mRNA encoding for the different subunits [108,109]. This led to other hypotheses, such as reduced receptor turnover, promotion of the assembly and migration to the plasma membrane of pre-existing intracellular subunits [110] or decrease in the rate of receptor turnover [111]. More recently, Sallette *et al.* [112] demonstrated that nicotine acts as a maturation enhancer (chaperone) of those intracellular nAChR precursors that would otherwise be degraded. However, different authors show controversial results. Vallejo *et al*. [113] reported that α4β2 up-regulation by nicotine is due to an increase/stabilization of the proportion of receptors in a high affinity state and not to an enhancement in receptor maturation.

Regardless of the mechanism, according to competition experiments demonstrating the affinity of METH and MDMA for nAChR, it could be hypothesized that the up-regulation of nAChR induced by these drugs would follow a similar mechanism than that of nicotine: binding to immature forms of the receptor inhibiting their degradation, promoting their migration to the plasma membrane or stabilizing the high-affinity state.

**Figure 7:**
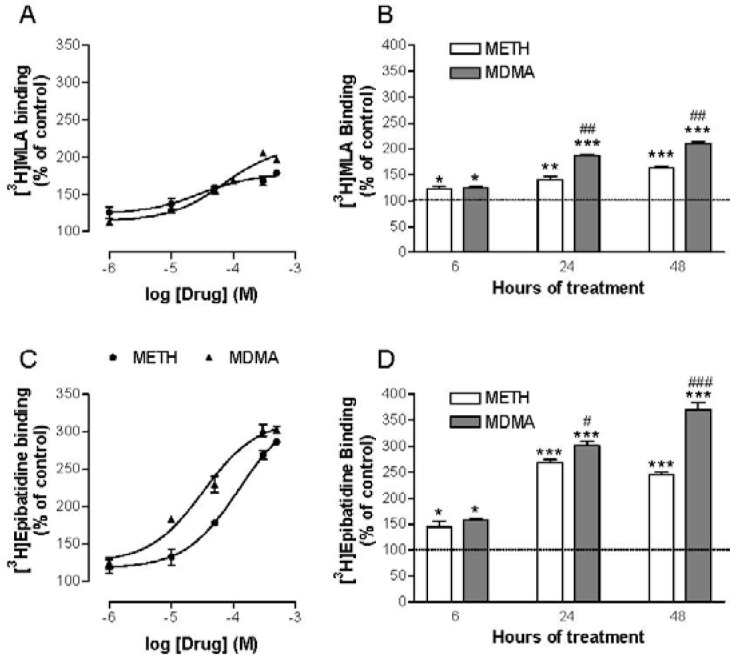
Concentration (**A**, **C**) and time (**B**, **D**) dependence of METH- and MDMA-induced nAChR up-regulation in differentiated PC12 cellls. PC12 cells were differentiated with NGF and incubated for 24 h at different concentrations of drug or for different times at a concentration of 300 μM. Panels **A** and **B** show the effects on homomeric α7 receptors and **C** and **D** those on heteromeric receptors.

Accordingly, it was tested whether METH and MDMA had any effect on α7 and heteromeric nAChR binding densities in PC12 cells and found that both were increased in a time- and concentration-dependent manner [103] (Figure 7). Additional experiments with selective inhibitors were performed in order to ascertain the underlying mechanism, pointing that METH and MDMA up-regulate nAChR through a complex post-transcriptional process but in a similar manner than nicotine. Moreover, the work done to date indicates that up-regulation can occur if the drug has a particular affinity to one or more nAChR subunits; regardless of its agonist/antagonist properties (*i.e.*, the antagonist DHBE is also able to induce it [114]). In addition, up-regulation is enhanced when the drug crosses the cell membrane to interact with immature forms of the receptor [115]. The affinity of METH and MDMA for both heteromeric and α7 nAChRs has been demonstrated and these drugs can reach the cytosplasm after transport through the dopamine transporter [116,117], which is abundant in PC12 cells. Therefore, the interaction of METH and MDMA with immature receptor subunits is feasible to induce such up-regulation.

Preliminary *in vivo* experiments also suggest that certain MDMA dosing schedules induce nAChR up-regulation in brain and potentiate the regulatory effects of nicotine [117].

## 10. Effects of METH and MDMA on nAChR Activation: Calcium and Electrophysiology Experiments

### 10.1. Acute Effects

Activation of nAChR in PC12 cells produces an increase in intracellular calcium, either directly (through α7 channels opening) or indirectly (after initial depolarization by heteromeric nAChR activation and opening of voltage-gated calcium channels) [118]. Garcia-Ratés *et al.* [119] used a fluorimetric method to investigate the effect of MDMA on Ca^2+^ levels in cultured PC12 cells and the involvement of different nAChR subtypes and other cell pathways related to Ca^2+^ mobilization [119].

**Figure 8:**
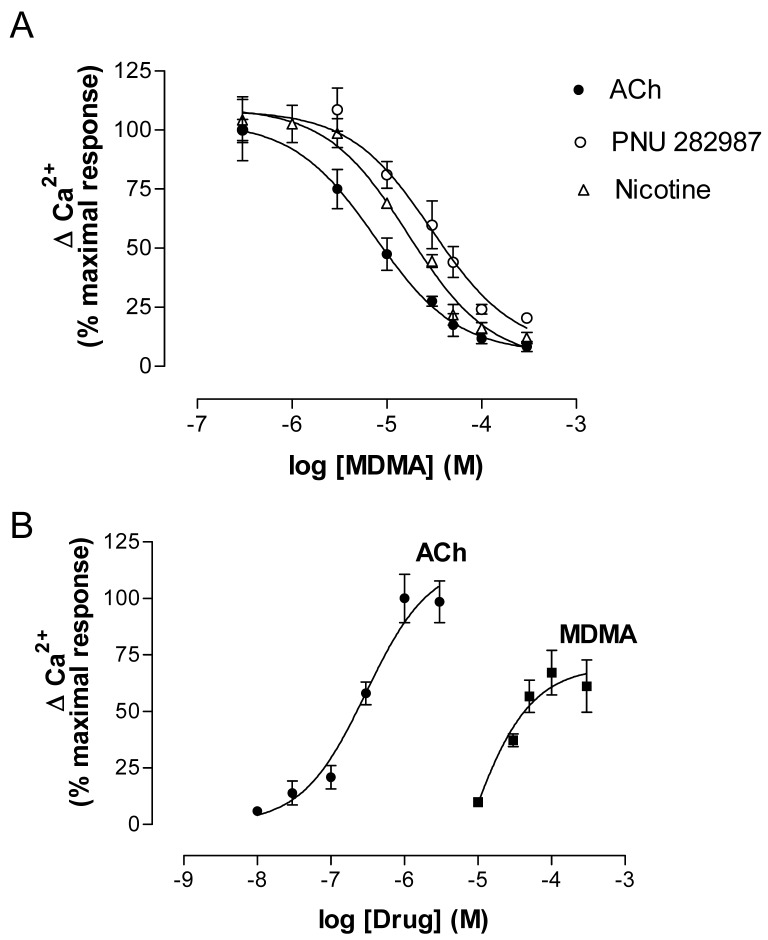
(**A**) Effect of increasing concentrations of MDMA on the responses to the nicotinic agonists ACh (100 μM), nicotine (100 μM) and PNU 282987 (0.1 μM) in PC12 cells loaded with Fluo-4. MDMA was added to the cells 5 min before the agonist. Basal fluorescence levels were measured for 5 s before the agonist and for a further 60 s after its addition by means of an automated injector. (**B**) Representative concentration-response curves showing the increase in cytosolic Ca^2+^ induced by MDMA and ACh as a total agonist. Responses were normalized as % (Fmax-Fmin) and represented as a percentage of the maximum response (ACh 100 μM) for both curves.

MDMA acutely inhibited the effects of nAChR agonists (ACh, Nicotine and PNU 282987) (Figure 8A) but, when applied alone at low micromolar concentrations, induced a concentration-dependent increase in Ca^2+^. The effect of MDMA did not reach the maximum values induced by ACh, which indicates a partial agonist mode of action (Figure 8). The EC_50_ value was around 45 μM, which is in agreement with previous binding results.

Electrophysiology experiments using *Xenopus* oocytes expressing human α7 and α4β2 nAChR also demonstrated an agonistic effect on α7 and an antagonist effect of MDMA on α4β2 nAChR [119].

The fact that MDMA induced an increase in cytosolic Ca^2+^ led to study the pathways involved using specific blockers. According to previous work [79], the α7 nAChR blockers MLA and α-bungarotoxin abolished the effect of MDMA, while the α4β2 antagonist dihydro-β-erythroidine did not modify it. Thus the increase in Ca^2+^ was initiated by activation of α7 nAChR and the binding affinity to α4β2 would be in agreement with the antagonist properties found in electrophysiology assays. A secondary implication of voltage-operated calcium channels and calcium-induced calcium release (CICR) from endoplasmic reticulum (ER) stores, which has been described to be coupled to α7 nAChR activation, was also found [37,118].

The MDMA response was dependent on extracellular Ca^2+^, as suppression of this cation totally inhibited its effect. Extracellular Ca^2+^ could enter through either α7 channels or L-type voltage-operated calcium channels and, as stated above, this Ca^2+^ increase would also induce subsequent CICR. Although mechanisms other than nAChR activation cannot be totally ruled out in the MDMA-induced increase in cytosolic Ca^2+^, the practically complete inhibition by MLA and α-bungarotoxin indicates that α7 nAChR activation plays a major role in this process.

### 10.2. Long Term Effects on Ca^2+^ Levels

As nAChR are desensitized upon sustained stimulation the effect of 24 h-incubation with MDMA on basal Ca^2+^ levels was studied [119]. MDMA induced an increase in basal cytosolic Ca^2+^ levels, measured after drug washout. Surprisingly, pre-incubation with nicotine only increased basal levels when it was carried out for 1 h, but not after longer pre-incubation times. This indicates that cells are able to buffer sustained activation by nicotine, but not that induced by MDMA, which suggests increased vulnerability to this drug as it allows continous Ca^2+^ entry leading to an excitotoxic-like process.

Sustained Ca^2+^ influx after MDMA could favor cytotoxicity through activation of Ca^2+^-dependent pathways (*i.e.*, calpain). Calpain is a calcium-dependent protease whose activation is a primary mechanism that contributes to several types of neurodegenerative conditions, including the excitatory amino acid-induced neurotoxicity that is associated with traumatic brain injury, ischemia, and hyperthermia [120,121]. Calpain specifically degrades the cytoskeletal membrane protein, spectrin, into 145 and 150 kDa breakdown products [122]. Caspase 3 is another cysteine protease that is involved in apoptotic pathways. It also degrades spectrin but produces a 120 kDa spectrin fragment [123] and also can produce a 150 kDa fragment [124].

**Figure 9:**
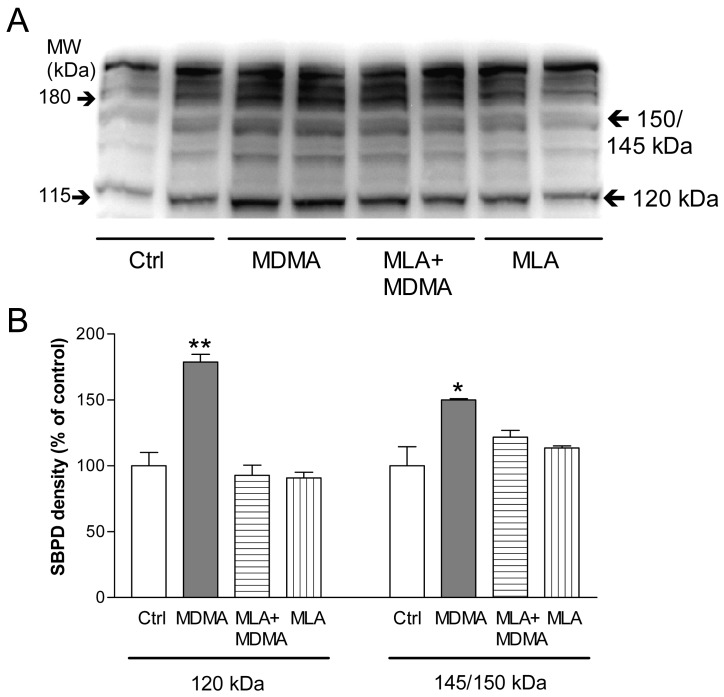
Representative Western blot of α-spectrin breakdown products (SBDP) originated by calpain activation (145 and 150 kDa) and capase 3 (120 and 150 kDa) after 24 h treatment with culture medium (Ctrl), MDMA (50 μM), MDMA + MLA (10 nM) and MLA alone. The localization of the molecular weight (MW) markers is shown on the left of the picture. B. Quantification of dot intensity of the SBDPs. Data are the means ± SEM of three different cultures, loaded in duplicates. * P < 0.05 and ** P < 0.001 *vs.* control. Reproduced from [119].

Incubation of PC12 cells with MDMA for 24 h induced a significant increase in spectrin breakdown products (SBDP) of 145 and 150 kDa, which indicates calpain activation, and a rise in the 120 kDa band that, together with the increase in the 150 kDa SBDP, points to caspase 3 activation (Figure 9). Moreover, the increases in SBDP induced by MDMA were prevented by MLA, indicating that α7 nAChR play a key role in this process.

### 10.3. Functional Up-Regulation

In addition to radioligand binding up-regulation, nAChR can suffer changes in stoichiometry and an increase in functional state (functional up-regulation) after prolonged incubation with a ligand [106]. Such up-regulation occurs also at a post-transcriptional level. When PC12 cells are incubated for 24 h with MDMA they exhibit increased responses to nicotinic agonists PNU 282987 (α7-selective) and 5-I-A-85380 (selective for β2 subunit-containing receptors), measured after drug washout [119]. This indicates that MDMA also induces functional nAChR up-regulation.

## 11. Concluding Remarks, Future Perspectives

Amphetamine derivatives are still a family of drugs with high incidence of abuse, mainly used with recreational and social purposes. Although they are believed to be safe by the users, there is clear evidence of cognitive impairment and dependence in frequent consumers [125]. In this review we have summarized a series of investigations that have added a new piece to the puzzle of the cellular effects of these drugs: the action on nicotinic receptors. An interesting point is the observation of prolonged calcium influx in cells (mediated by α7 nAChR) induced by MDMA, which leads to the activation of cytotoxic pathways and could account for long-term neurotoxic effects in frequent abusers. On the other hand, despite the antagonist effect on heteromeric nAChR, the regulatory effect of that these drugs exert on nAChR densities could be responsible for certain neuropsychiatric disorders.

One of the most interesting applications might be the possibility of preventing underirable effects, as demonstrated by the neuroprotection experiments. When it comes to a drug of abuse, the most advisable way of preventing undesirable effects is avoiding its intake. However, this new mechanism could provide additional strategies to treat or ameliorate the addiction or some of the deleterious effects caused by these drugs.

Memantine has shown promising results in the treatment of amphetamine addiction [126]. No drugs are currently approved in the U.S. or Europe for the treatment of addictions to METH or MDMA. Fluoxetine pre-treatment has been recommended as protection from MDMA-induced long term neurotoxicity, but recently it has been found that fluoxetine decreases the clearance of MDMA and its metabolite, methylenedioxyamphetamine, leading to an increased risk of acute MDMA toxic effects [127].

MDMA was formerly used as a tool in psychotherapy, but its undesirable effects led to its withdrawal. Also, it has been assayed and proposed as a drug to treat anxiety disorders including post-traumatic stress [128]. The availability of a neuroprotective treatment could lead to reconsider or facilitate such applications.

## Figures and Tables

**Figure 3:**
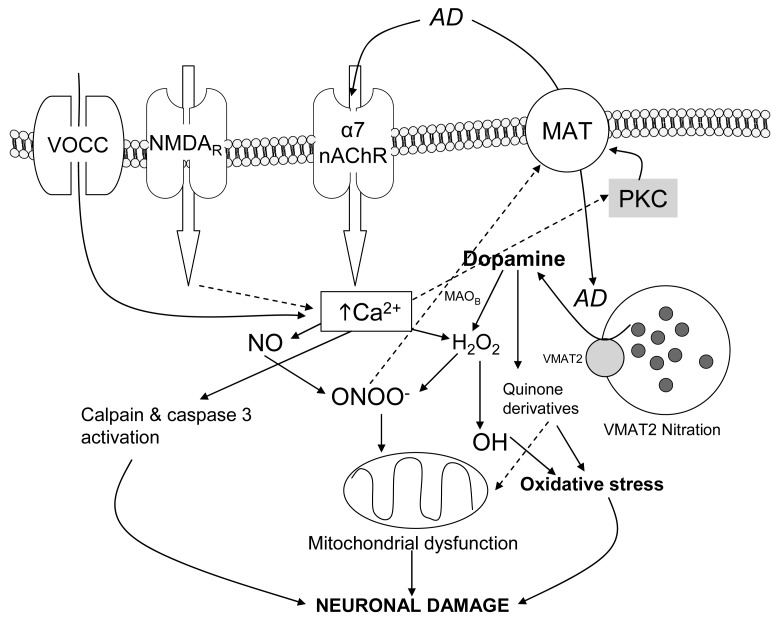
Schematic representation of the pathways involved in oxidative stress and neurotoxicity induced by amphetamine derivatives (AD). Activation of α7 nAChR by AD contributes, together with other proposed pathways (*i.e.*, NMDA receptor activation [61,71]), to a raise in intracellular Ca^2+^ which results in activation of nNOS and production of NO. NO can produce ONOO^−^ by reacting with superoxide. ONOO^−^ induces direct damage to cell structures and induces mitochondrial dysfunction leading to metabolic impairment and cytotoxicity. Also ONOO^-^ induces nitration of the plasmalemmal and vesicular monoamine transporters (MAT and VMAT, respectively). Ca^2+^ also activates calpain and caspase 3 (see section 7.2), which are involved in proteolysis and cell death mechanisms, as well as PKC, which is related to MAT blockade trapping DA inside the terminal. At the same time, AD induce increase in free intracellular DA that can be metabolised by MAO_B_ to H_2_O_2_ and superoxide which in turn can be further converted via the Fenton reaction to hydroxyl radicals that contribute to oxidative stress. Also dopamine can be metabolised to reactive quinone derivatives which contribute to cell function impairment [60]. Finally additional cytosolic Ca^2+^ can enter after depolarisation through voltage-operated calcium channels (VOCC) or can be released from the endoplasmic reticle.

**Figure 4:**
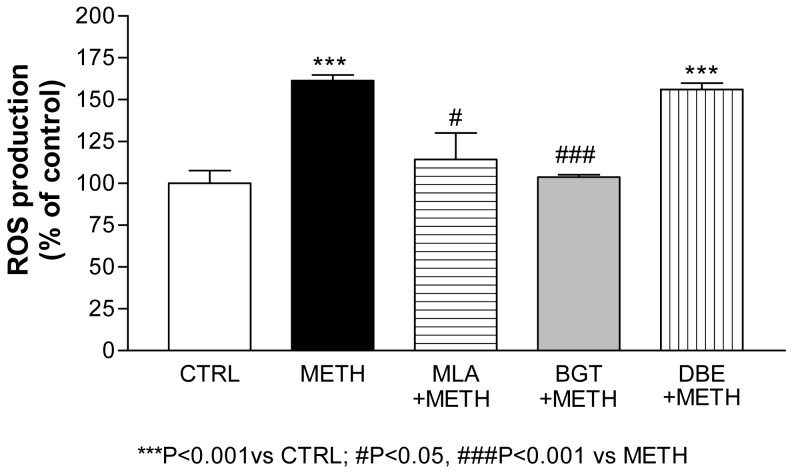
ROS induction by METH in striatal synaptosomes and its inhibition by α7 nAChR antagonists [29] (MLA and α-bungarotoxin, BGT). The α4β2 antagonist dihydro-β-erythroidine (DBE) was devoid of effect.

**Table 1:** K_i_ values of METH and MDMA against [^3^H]MLA and [^3^H]epibatidine binding in PC12 cells and mouse brain. n_H_ is the Hill coefficient. * *P* < 0.05 and ** *P* < 0.01 *vs.* 1 (one sample t test). Data reproduced from [103].

	**PC12 cells**		**Mouse brain**	
**Drugs**	**K_i_(μM)**	**n_H_**	**K_i_(μM)**	**n_H_**
	[^3^H]MLA			
**METH**	283 ± 109	1.20 ± 0.15	369.77 ± 95.61	0.29 ± 0.08 **
**MDMA**	15.35 ± 1.03	1.35 ± 0.11	34.21 ± 6.71	0.40 ± 0.27 *
	[^3^H]Epibatidine			
**METH**	155.36 ± 5.36	0.76 ± 0.11	23.90 ± 2.65	1.27 ± 0.28
**MDMA**	25.71 ± 3.13	0.92 ± 0.04	0.76 ± 0.11	0.83 ± 0.12
